# Training Teachers for Self-Regulated Learning: A Structured Narrative Review

**DOI:** 10.3390/ejihpe16040055

**Published:** 2026-04-20

**Authors:** Lucía Poladura, Elena Blanco, Ellián Tuero, Celestino Rodríguez, José Carlos Núñez

**Affiliations:** Department of Psychology, University of Oviedo, 33003 Oviedo, Spain; poladuralucia@uniovi.es (L.P.); blancoelena@uniovi.es (E.B.); tueroellian@uniovi.es (E.T.); jcarlosn@uniovi.es (J.C.N.)

**Keywords:** self-regulated learning, SRL, teacher training, pre-service, in-service teacher, professional development

## Abstract

This structured narrative review aimed to synthesize the findings of various studies to determine the efficacy of Self-Regulated Learning (SRL) training programs for in-service and pre-service teachers on their knowledge and skills, and to evaluate the transfer to teaching practice and student outcomes. Following PRISMA guidelines, a search was conducted across Web of Science, Scopus, and PsycInfo, ultimately including 30 intervention studies. The results confirmed that professional development is effective in enhancing teachers’ knowledge, skills, and beliefs related to SRL. However, due to wide methodological diversity, the review identified varied intervention factors showing promise, but a unified association between sample type (in-service vs. pre-service) and overall impact was unattainable. While SRL training successfully improves teacher competency, the limited evaluation of student performance or long-term effects prevents the definitive claim that the training reliably changes teaching practice toward a more self-regulated approach. Future research should prioritize robust longitudinal designs and include student-level measures.

## 1. Introduction

The primary aim of Self-regulated learning (SRL) is to foster autonomous students who can regulate their emotions, actions, and thoughts when pursuing the academic goals they have set themselves (Panadero & Alonso-Tapia, 2014). Over the years, various theories have emerged to explain this type of learning, ranging from the phases it goes through to the internal and external factors that can influence the process (Boekaerts, 1997; Pintrich et al., 2000; Zimmerman, 2000).

One of the most widely studied, influential theories is Zimmerman’s Cyclical Model of Self-Regulated Learning (Zimmerman, 2000), which describes learning as a cyclical process that connects metacognitive and motivational processes. As (Panadero, 2017) explained, the theory is structured into three main phases, forethought, performance, and self-reflection, which all students have to go through when dealing with a new task.

In the forethought phase, students engage in task analysis, set their own learning goals, and plan the most appropriate strategies to achieve those goals. Self-motivational beliefs play a key role, and are influenced by five variables: self-efficacy, expectations, task value, interest, and goal orientation. The performance phase consists of two sub-phases: self-observation and self-control. In self-observation, students monitor their progress with the task, comparing it to their initial plan. In self-control, they maintain concentration using metacognitive and motivational strategies. Finally, in the self-reflection phase, students evaluate both the task outcome and the process they followed through self-evaluation and causal attributions that produce self-judgments. These directly influence self-reactions, which manifest in satisfaction with one’s work and willingness to repeat similar tasks. Emphasizing the cyclical nature of this theory, the self-reflection phase is fundamental in the learning process because the results directly impact the forethought phase of the next task. Consequently, self-judgments and self-reactions prepare students either to replicate the same planning if the result was satisfactory, or identify a need to adjust objectives and use different strategies if it was not. Altogether, this produces a model in which the three phases mutually reinforce one another, forming a continuous loop throughout the learning process.

The importance of SRL has led to numerous interventions designed to enhance SRL competencies in classrooms, fostering student autonomy, academic achievement, and overall well-being (Chu et al., 2020; Valiente-Barroso et al., 2020) across all educational stages and in various fields of knowledge (de Boer et al., 2018; Dignath-van Ewijk & Büttner, 2018; Theobald, 2021). Zimmerman (2000) stated that good self-regulated learners start with good self-regulated teachers. To achieve this, SRL needs to be brought closer to teachers. Nevertheless, there appears to be a gap between the knowledge and terminology related to this concept in teaching and in research, meaning that many teachers do not know how to apply SRL in the classroom or are not confident in their abilities to teach these types of strategies to students (Callan & Shim, 2019; Latva-aho et al., 2024).

Teachers seem to associate self-regulated learning with self-directed learning—where students take the initiative and do not require any kind of teacher support. Although these concepts are potentially related, they are not synonymous (Callan & Shim, 2019). When it comes to identifying self-regulating elements during the learning process, teachers do not fully comprehend how they should support students’ development of these skills (Callan & Shim, 2019; Latva-aho et al., 2024).

Several studies have demonstrated difficulties with identifying the phases of the self-regulatory process and particularly when defining the constituent elements and concepts. This is true not only for active teachers but also for future teachers, who also appear to find this task challenging (Callan & Shim, 2019; Dignath-van Ewijk & van der Werf, 2012; Latva-aho et al., 2024). In this regard, (Baumert & Kunter, 2013) suggested that teachers’ professional practice is a result of the interaction of several factors, including professional values, beliefs and goals, motivation, and the teacher’s self-regulatory skills. Therefore, most of the studies present teacher training as a good alternative to reach a consensus in the SRL field between teachers and investigators, and effectively promote students’ SRL through teachers’ professional practice (Dignath-van Ewijk & van der Werf, 2012).

### 1.1. Teacher Training for Self-Regulated Learning

Training and professional support can change teachers’ misconceptions and false beliefs about self-regulated learning (SRL). This additional training improves their self-efficacy, allowing them to feel capable and confident when identifying and implementing all the phases and components of SRL in the classroom (Latva-aho et al., 2024; Perry & VandeKamp, 2000). Darling-Hammond et al. (2017) defined effective training as the activities that teachers undertake, either informally outside the institution or as part of their professional duties, which help them modify their knowledge and educational practices to improve students’ learning outcomes and support their individual learning processes.

The impact of professional development on teachers’ knowledge, skills, and beliefs has been demonstrated across various areas of expertise (Donath et al., 2023; Kalinowski et al., 2019). For instance, in inclusive education, Brock and Carter (2017) highlighted that training contributes to improving teaching practice with students with disabilities, provided that certain methodological guidelines—such as modeling or feedback—are considered. Similarly, Donath et al. (2023) sought to confirm that training not only improved participating teachers’ practice but also led to observable changes in teachers’ beliefs, knowledge, and skills. They argued that intervention with teachers can increase knowledge and beliefs about effectively implementing inclusive education in the classroom (self-efficacy). However, these changes were more modest when evaluating actual performance, due to obstacles the teachers themselves perceived. The authors concluded that changing beliefs may be more complex than changing their knowledge or skills, as they are influenced by not only knowledge and positive experiences, but also by institutional culture and personal values.

In a study focused on a specific domain of knowledge, Siegle and McCoach (2007) examined an intervention to train teachers from ten different schools in the use of self-efficacy strategies during mathematics classes. The results indicated that participants increased their knowledge of these strategies, and the teachers reported feeling capable and confident in applying them in the classroom. This study supports the research that teachers can modify their classroom practice with minimal training, which can in turn influence students’ efficacy and learning outcomes. Similarly, Amemasor et al. (2025) found in their review that teacher training aimed at integrating digital instruction in classrooms proved effective in changing teachers’ mindsets and attitudes, provided that the training programs addressed the specific needs of the group and considered the broader implementation context and available infrastructure. Along similar lines, Kalinowski et al. (2019) focused on the development of academic linguistic competence. Following a review of 38 articles, they concluded that there is considerable methodological and implementation diversity in training programs. Although all of the included studies demonstrated some type of positive impact, it was difficult to determine which factors were key to successful interventions.

### 1.2. Training Formats and Results

Professional development initiatives—delivered through diverse modalities such as face-to-face, online, and hybrid formats—have been designed to strengthen teachers’ SRL-related knowledge, beliefs, and pedagogical practices (Stavermann, 2024). In this regard, Sankar and Sankar (2010) implemented a teacher training program on co-teaching competencies using two intervention formats—face-to-face and online. The results showed that both formats contributed to improving teachers’ knowledge of teamwork, co-teaching, and teaching responsibilities. However, only the face-to-face program was capable of increasing participants’ confidence in these new skills. Both formats have advantages. Online implementation can help knowledge acquisition by allowing participants to complete training at their own pace, helping them consolidate and reflect on newly acquired learning (Guskey, 2002). However, if content is particularly complex, participants may benefit more from an in-person format, where instructor and peer support can help boost self-confidence (Sankar & Sankar, 2010).

More specifically, Sankar and Sankar (2010) observed that teachers sometimes prefer face-to-face to asynchronous formats. However, in one of the most recent reviews, Stavermann (2024) found generally positive effects on teachers’ knowledge, skills, and practice after online or hybrid training, along with high levels of satisfaction with these formats. Factors ensuring the success and effectiveness of this type of training include flexibility, instructor support, and opportunities for collaboration and interaction among participants (Lay et al., 2020; Stavermann, 2024).

In summary, comparative evidence across modalities suggests that while each format contributes to teacher learning, blended formats tend to yield the most consistent improvements in both knowledge and classroom implementation (Bautista & Ortega-Ruíz, 2015; Darling-Hammond et al., 2017). In-person formats show strong short-term gains in confidence and skill demonstration, whereas online and hybrid models support longer-term retention and autonomy (Lay et al., 2020). Nevertheless, sustained support and follow-up coaching remain critical for maintaining changes in teachers’ beliefs and practices (Desimone & Garet, 2015; Kyndt et al., 2016). Across studies, professional development in SRL is positively correlated with teaching quality, teachers’ capacity to promote student metacognition, and overall student achievement (Dignath-van Ewijk & Büttner, 2018; Perry et al., 2008).

### 1.3. Present Study

Overall, the literature from the last twenty years underscores the idea that teacher training in SRL—whether delivered in-person, online, or through blended methods—enhances educators’ understanding, self-efficacy, and instructional competence. The most effective programs integrate active engagement, feedback, collaboration, and contextual relevance. However, further research is needed to determine how long these effects last and the mechanisms by which SRL-oriented professional development translates into sustained classroom transformation. Therefore, the present structured narrative review seeks to address this gap through the collection and synthesis of findings from various conducted to date, providing an overview of the current state of the relationship between teacher training in SRL and the development of skills and knowledge associated with this type of learning. To that end, four research questions are proposed:

RQ1. What are the distinctive characteristics (format, duration, methodology, etc.) of the SRL training programs that have been implemented?
RQ2. How does such training increase teachers’ knowledge of self-regulated learning?
RQ3. How does the teacher training in self-regulated learning subsequently reflected in their classroom practice and in their students?
RQ4. How does the training affect the long-term teaching practices of the participants?

## 2. Materials and Methods

### 2.1. Information Sources and Search Strategy

The search was conducted from 6 March–31 March 2025 across three databases simultaneously: Web of Science (WoS), Scopus, and PsycInfo. During an initial exploratory phase, multiple searches were performed using various keywords and combinations of the terms. However, these did not yield satisfactory results. Consequently, the search strategy was refined, opting for a single, more precise search string, structured using Boolean operators. The final search equation was as follows: (“self-regulated learning” OR “self-directed” OR “SRL” OR “self-regulated strateg*”) AND (“teacher training program” OR “teacher training course” OR “teacher education” OR “teacher professional development”).

The first general search was conducted without filtering the published year of the manuscripts and considering only those written in English. Since the largest number of publications were found within the last ten years, to narrow the results it was decided to limit the search to this period since the studies published before that were only few and outdated.

### 2.2. Inclusion and Exclusion Process

The SPIDER format (Sample, Phenomenon of interest, Design, Evaluation, Research type) was used to define the eligibility criteria, which allows systematic structuring of the key elements (Sánchez-Martín et al., 2023). The specific inclusion and exclusion criteria are detailed in Table 1.

### 2.3. Study Selection Process

The study selection process followed a series of sequential phases, adhering to the PRISMA guidelines (Page et al., 2021). Mendeley was used for reference management and identification of duplicates. A PRISMA Checklist is provided as Supplementary Material.

**Figure 1:**
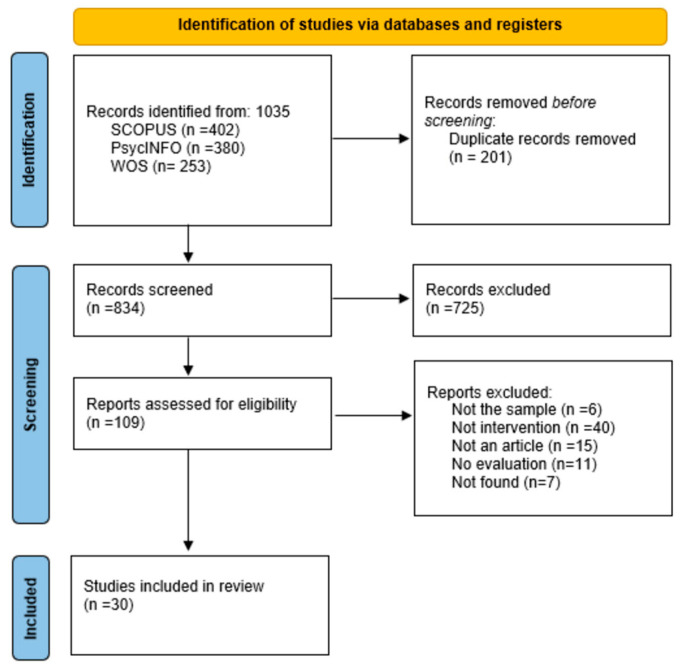
PRISMA Flow Diagram.

The study search and selection process yielded a total of 1035 records, 402 from Scopus, 380 from PsycINFO, and 253 from Web of Science. As detailed in the PRISMA flow diagram (Figure 1), the records were subjected to a multi-phase screening process: identification, screening, and eligibility. In the identification phase, 201 duplicate records were eliminated. In the screening phase, the titles and abstracts of the remaining records (n = 834) were reviewed according to the inclusion and exclusion criteria, resulting in a reduction to 109 records. Finally, in the eligibility phase, the full texts were evaluated, and 79 studies were excluded for not meeting the inclusion criteria. The main reasons for exclusion were: the sample did not consist of in-service or pre-service teachers, the main focus was not SRL training, or there were no intervention or program evaluation studies. Following this process, 30 manuscripts were selected for inclusion in the structured narrative review.

First, duplicate records identified across the three databases were removed. The articles were then screened, with titles and abstracts evaluated according to the inclusion and exclusion criteria. Finally, shortlisted articles were examined in more detail, which involved an in-depth appraisal of the method and results sections. In this stage, the inclusion and exclusion criteria were applied again, since some of the studies did not make clear their sample or characteristics on their abstract. Last step involved the discarding of studies that did not align with the research questions.

This process of identifying studies was conducted independently by two researchers. The concordance between them in the selection of the full texts was assessed by calculating Cohen’s Kappa coefficient (κ), yielding a value of 0.90, which indicates almost perfect agreement (Landis & Koch, 1977). When initial agreement was not achieved, a meeting was held to analyze the reasons for the discrepancy and reach a final joint decision. Both authors presented their reasons for accepting or rejecting an article, and the studies where there was no consensus were analyzed in detail, considering the established inclusion and exclusion criteria, to justify the final decision. In the event of a persistent disagreement, a third external and objective expert was consulted.

### 2.4. Quality Assessment

A methodological quality analysis was performed on the final sample of 30 studies, based on the seven Methodological Quality Indicators (MQIs) proposed by Miller et al. (2016). For the purposes of this review, the MQIs were applied as a descriptive-exploratory to identify methodological patterns and tendencies across the corpus, rather than as a rigid evaluative system. Within this framework, the assessment addressed seven. dimensions: research objectives, theoretical–empirical alignment, methodological description, reliability, validity, participants characteristics, and the adequacy of findings (Table 2).

Following the methodology of Camacho et al. (2021), manuscripts were scored on a binary scale (1/0), where the total score of 6 or higher indicated high methodological quality.

To ensure reliability, the assessment was conducted by one researcher and independently validated by a second. No ambiguous cases arose during the appraisal process, as both researchers reached full agreement on all evaluations. All studies achieved the required quality standards and were included in the final synthesis (Appendix A).

### 2.5. Data Extraction Process

Data extraction was performed using Microsoft 365 tools, specifically Excel and Word. This process was carried out by one researcher and then reviewed and validated by a second researcher. All selected manuscripts were analyzed, and the relevant information to answer the research questions was extracted. The areas of interest that the extracted data focused on were: general characteristics of the paper, general characteristics of the training program, and evaluation of the training program (see Table 3).

The data was initially extracted into summary tables in a Word document and then coded in Excel for categorization (Appendix B). For greater standardization, the results were evaluated using a rubric that was designed based on the concept of professional competence defined by Baumert and Kunter (2013). This rubric functioned as a descriptive and exploratory tool aimed at outlining the impacts identified in the interventions. In line with this structure, intervention programs were scored on a scale of 0 to 3, where 0 indicated “no impact” and 3 represented a “high impact” The categories evaluated included: knowledge and skills (“improvement of knowledge on the topic” and “knowledge on how to intervene”), beliefs (“changes in beliefs” and “conceptions about self-regulated learning”), motivation (“increase in interest” and “perception of benefits”), and SRL (“increase in self-efficacy”). The scores were given by looking at the results of each intervention and seeing if they presented highly or not significant results for the categories established. Not all elements of the rubric were applicable to all reviewed articles. In such cases, a “not applicable” option was included to reflect the relevance of each criterion to the nature of the evaluated study (Appendix C).

### 2.6. Study Synthesis Process

The results of the studies were synthesized via qualitative review due to the heterogeneity of the study designs, intervention characteristics, and samples (Aguilera, 2014). The qualitative synthesis was structured around the four research questions, allowing for detailed description of the findings. Heterogeneity was addressed by grouping the articles based on whether they focused on in-service or pre-service teachers. Results were presented descriptively through text, complemented by figures for the fourth research question.

### 2.7. Bias Assessment

During the structured narrative review process, guided by the PRISMA protocol (Page et al., 2021), special emphasis was placed on assessing and minimizing potential biases. To that end, both the search strategy and the inclusion and exclusion criteria were reviewed by several researchers, including experts in the field of teacher training in self-regulated learning and new technologies. Furthermore, as previously explained, multiple researchers participated in study identification and data extraction. Finally, to evaluate publication bias, a transparent and systematic approach was adopted during the search. An exhaustive search strategy across multiple databases was implemented to reduce the probability of omitting relevant studies and the potential impact of the non-publication of results.

## 3. Results

The 30 identified studies measured the increase in knowledge about the elements of self-regulated learning. These studies focused on both in-service teachers (n = 14) and student (pre-service) teachers (n = 16) in various knowledge areas. Furthermore, 10 of the studies extended their scope to evaluate whether this new knowledge manifested in teaching practice through teachers incorporating self-regulation in the classroom. This practical manifestation was examined using analysis of curricula, field diaries, videos of actual classes, direct observation, or responses to questionnaires and interviews. However, out of the 30 studies, only a few (n = 4) considered the impact of the training on students and even fewer (n = 3) conducted follow-ups to determine whether the effects of the intervention continued in the long term.

RQ1. What are the distinctive characteristics of the SRL training programs (format, duration, methodology, etc.) that have been implemented?

To describe the characteristics of the selected studies (pedagogical approach, methodology, and duration) they were grouped into two main categories based on the intervention samples: programs targeting in-service teachers and programs targeting pre-service (student) teachers (see Appendix B).

Programs Targeting In-Service Teachers (n = 14)

The majority of these programs were based on Zimmerman’s Cyclical Model of Self-Regulated Learning (n = 10) (e.g., Arcoverde et al., 2020; Inan-Karagul & Seker, 2021). Other studies (n = 3) combined this model with other relevant theories, such as those from Pintrich and Boekaerts (e.g., Dignath, 2021; Kramarski & Kohen, 2017). The remaining study focused on a less-widely studied model in the scientific literature (Kaemper et al., 2024).

Each program had its own objectives and intervened in various fundamental elements in the self-regulation process. The majority (n = 11) adopted a holistic view of the process, providing training that covered all phases (e.g., Linde et al., 2023; Peters-Burton et al., 2024). A minority (n = 3) mainly focused on teachers’ self-reflection process (e.g., Ambinintsoa & Castro, 2024; Bruna et al., 2023), which is fundamental during practice.

There was a wide range of intervention methodologies. Primarily, there were courses following expository or lecture-based formats (n = 6) (e.g., Bruna et al., 2023; Porter & Peters-Burton, 2021), followed by learning communities (n = 3) (Adigüzel et al., 2023; Barr & Askell-Williams, 2020; Xu & Ko, 2019), and the use of digital tools specifically designed for the intervention (n = 2) (Peters-Burton et al., 2024; Sins et al., 2024). Other studies (n = 3) used more specific methodologies such as learning journals (Ambinintsoa & Castro, 2024), training through videos (Heaysman & Kramarski, 2022), and even a board game (Persico et al., 2023). Most of these interventions (n = 10) were conducted in face-to-face settings (e.g., Barr & Askell-Williams, 2020; Cleary et al., 2022), while others were online (n = 3) (Adigüzel et al., 2023; Linde et al., 2023; Sins et al., 2024) or hybrid (n = 1) (Peters-Burton et al., 2024).

Coding the duration was more challenging because of the wide range of time units used in each study. Several (n = 6) indicated that the intervention was carried out over several weeks (ranging from 1 to 32) (e.g., Bruna et al., 2023; Porter & Peters-Burton, 2021), while others (n = 5) detailed the number of hours, ranging from 6 to 30 (e.g., Xu & Ko, 2019; Heirweg et al., 2022). Only the study by Persico et al. (2023) mentioned a specific duration of three days, and the other (n = 2) corresponded to longitudinal studies by Linde et al. (2023), which lasted for one year, and Peters-Burton et al. (2024) intervention that was 3 years long.

Therefore, interventions targeting in-service teachers show several recurring tendencies. Most programs are grounded in Zimmerman’s cyclical model and adopt a holistic perspective of self-regulation, combining conceptual input with opportunities for reflection on teaching practice. Despite the heterogeneity in delivery formats and duration, face-to-face courses remain the predominant structure, and interventions tend to be relatively short. These features collectively suggest that, in the current literature, professional development for in-service teachers is primarily approached through compact, theory-driven programs aimed at providing an overarching understanding of SRL rather than deep, long-term practice-based training. 

Programs Targeting Pre-Service Students (n = 16)

These programs exhibited a wider range of reference theoretical models. Zimmerman’s theory (n = 9) remained predominant (e.g., Arcoverde et al., 2020; Ganda & Boruchovitch, 2018), followed by Boekaerts’ model (n = 2) (Michalsky, 2014, 2020), the Zurich Resources Model (Kaemper et al., 2024), and a combination of the Zimmerman and Pintrich models (Kramarski & Kohen, 2017). Three studies did not specify a theory (e.g., Hursen & Fasli, 2017; Romig et al., 2018).

In terms of intervention methodology, lectures were again the main approach (n = 6) (e.g., Stephenson et al., 2024; Tran et al., 2022), followed by digital tools (n = 4) (e.g., Kaemper et al., 2024; Ortega-Ruiperez et al., 2024) and videos with real classes for student training (n = 3) (Inan-Karagul & Seker, 2021; Kramarski & Kohen, 2017; Michalsky, 2020). Other studies (n = 3) used specific methodologies such as learning journals (Dignath-van Ewijk et al., 2015), focus groups (Arcoverde et al., 2020), and even podcasts (Romig et al., 2018). Most of these interventions targeted development of general knowledge in self-regulation (n = 13) (e.g., Ganda & Boruchovitch, 2018; Tran et al., 2022). Only (n = 2) Hursen and Fasli (2017) and Kaemper et al. (2024) focused the training on the reflection process, while Michalsky (2014) focused solely on fostering metacognition.

Almost half of the interventions were face-to-face (n = 7) (e.g., Kramarski & Kohen, 2017; Zeeb et al., 2024), the same number of studies delivered the interventions online (n = 7) (e.g., Barz et al., 2024; Dignath-van Ewijk et al., 2015), and only the intervention in Ortega-Ruiperez et al. (2024) was delivered in a hybrid manner.

Similar to the interventions targeting in-service teachers, standardizing duration was complex. Only Ganda and Boruchovitch (2018) and Stephenson et al. (2024) specified the specific number of hours, while most studies (n = 10) chose to count the intervention in weeks, ranging from 2 to 14 weeks (e.g., Inan-Karagul & Seker, 2021; Michalsky, 2024). Kramarski and Kohen (2017) and Zeeb et al. (2024) counted the number of seminars held, and the rest (n = 2) noted durations in days (Romig et al., 2018) or did not specify, like Ortega-Ruiperez et al. (2024).

Overall, the pre-service teacher programs display greater theoretical diversity and make more frequent use of digital tools and video-based approaches. These interventions typically emphasize foundational SRL knowledge, with fewer programs focused on reflective processes or advanced application. Delivery formats are evenly distributed between face-to-face and online modes, and durations vary considerably, although most fall within short- to medium-term ranges. Overall, these tendencies suggest that SRL training in initial teacher education is predominantly conceptual, supported by technology-enhanced instructional methods, and designed to fit within standard program structures.

RQ2. How does such training increase teachers’ knowledge of self-regulated learning?

**Figure 2:**
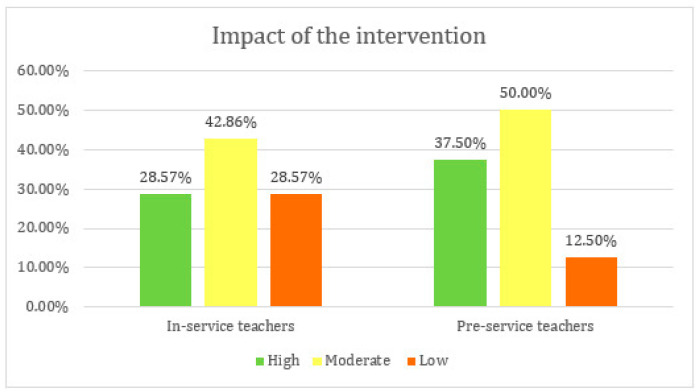
Impact of the intervention programs based on the target population.

To standardize the results from the different studies and make them easier to compare, the evaluation rubric described in the Methodology section—based on Baumert and Kunter’s (2013) professional competence model—was applied. Figure 2 shows that 28.6% of the training programs with in-service teachers had a high impact, rising to 37.5% in pre-service interventions.

The heterogeneity in training approaches and goals makes it challenging to directly compare the impact of the interventions. When classifying the manuscript by the type of intervention sample, there was no consistent pattern in the level of outcomes achieved. The pre-service sample exhibited a broad distribution of outcomes (high positive impact n = 6; moderate n = 8; low n = 2) that was similar to the distribution in the in-service sample (high n = 4; moderate n = 6; low n = 4). The variability of the findings suggests that the success of the interventions depended on factors beyond mere sample type.

Nevertheless, a comparison of the study outcomes (high vs. moderate/low) based on intervention sample was conducted using Fisher’s exact test, as the expected count for one of the cells was below 5. The results indicate that there was no statistically significant difference in the percentages of positive outcomes between the two groups (Fisher’s exact test, *p* = 0.709). Consequently, no association can be demonstrated between the study sample and the intervention outcome.

The programs that had a high impact included interventions reported by Adigüzel et al. (2023), Barr and Askell-Williams (2020), and Xu and Ko (2019), which used collaboration among teachers as a training method, especially through learning communities. Similarly, Inan-Karagul and Seker (2021), Kramarski and Kohen (2017), and Michalsky (2020) reported positive results, all using videos of real classes either as the main methodology or as support in training student teachers about the self-regulation process. However, this pattern was not replicated in the study by Heaysman and Kramarski (2022) with in-service teachers. Despite using videos and simulations, they reported a moderate impact.

Taken together, the findings on program impact do not show a consistent pattern linked to the participant group. Instead, studies reporting stronger effects often share methodological features such as collaboration-based structures or the incorporation of video analysis, which appear beneficial across both in-service and pre-service contexts. At the same time, variation within each category indicates that these features are not universally predictive of higher impact.

RQ3. How does the teacher training in self-regulated learning subsequently reflected in their classroom practice and in their students?

We expected that more studies would have evaluated the impact of teacher training on their students. However, only four (Adigüzel et al., 2023; Dignath, 2021; Heirweg et al., 2022; Xu & Ko, 2019) included evaluations of student outcomes, and there was a wide range of forms of evaluation and results from those programs. The studies by Adigüzel et al. (2023) and Xu and Ko (2019) reported increases in students’ self-regulation skills. The studies assessed these skills via materials students produced during the application of the teachers’ lesson plans (Adigüzel et al., 2023) and learning journals (Xu & Ko, 2019). In contrast, the intervention by Heirweg et al. (2022) produced a slight or mild effect on student knowledge, measured through questionnaires. Finally, Dignath (2021), who used a written test to evaluate students, found no significant changes in the students’ use of self-regulatory strategies.

All things considered, the limited number of studies evaluating student outcomes reveals some tentative patterns. Interventions that incorporate student-generated artifacts or reflective products tend to report clearer improvements in SRL, whereas those relying solely on questionnaire-based measures find more modest or inconsistent effects. The small number of studies and their methodological heterogeneity constrain the strength of these observations.

RQ4. How does the training affect the long-term teaching practices of the participants?

Of the 30 interventions included in this review, only three (10%) incorporated long-term measures allowing confirmation of real acquisition and use of the skills and knowledge about the various elements of self-regulated learning the teachers were trained in.

Ambinintsoa and Castro (2024) conducted a follow-up through interviews with two participants of their program, two years after the end of a course focusing on reflection and self-evaluation. Both students stated that they continued to use the strategies learned during the course, as well as having transferred them to other learning subjects and even to tasks in their daily lives. Michalsky (2020) conducted a follow-up one semester after having implemented her program with student teachers. The results indicate that the impact of direct and indirect teaching of self-regulation strategies was maintained over time and improved without any additional instruction. Lastly, Stephenson et al. (2024) conducted a follow-up six weeks after their course finished. They confirmed that the effect of the training on teaching self-regulation to future teachers was maintained.

## 4. Discussion

The primary objective of this review arose from the need to determine whether training programs for teachers about self-regulated learning are effective in increasing their knowledge and skills, and whether the results of such training can influence professional activity and student performance.

The results, limited by the search strategy, the exploratory tools used for their synthesis, and the heterogeneity of the studies, do not aim to provide definitive proof but rather suggest that certain methodologies may be more conducive to support the effectiveness of professional development in enabling both in-service and pre-service teachers to complete and expand their knowledge related to self-regulated learning. Although there was no clear relationship between certain elements and high effects due to the wide range of methodologies and ways that training was implemented, the majority of the interventions improved teachers’ knowledge, skills, and beliefs. These results will be discussed based on three dimensions: 1. Impact of teacher collaboration, 2. Factors influencing effectiveness, and 3. Duration of training and progress measurement.

Firstly, the identified patterns of interventions using teacher collaboration as a training method, through learning communities or communities of practice, suggest that it could be more effective. Adigüzel et al. (2023) approached their self-regulation training from a modeling perspective but adopted the learning community as their methodology, emphasizing communication between participating teachers, debating ideas, and especially feedback from experts before and after teachers implemented their lesson plans. Similarly, Barr and Askell-Williams (2020) created a community where teachers shared ideas, experiences, and solved problems among themselves, culminating in the design of a self-regulatory lesson plan. Both experiences highlight the importance of collaboration, communication, and feedback, which are fundamental elements of a learning community. There is ample literature supporting the efficacy of this type of methodology in teacher training. Authors such as Lipowsky and Rzejak (2015) and Desimone (2009) stated that collaboration between teachers has a positive impact on student performance and that the feedback they receive during idea exchange helps them identify changes they need to make in their teaching.

Similarly, Xu and Ko (2019) chose to implement a community of practice. While learning communities are created as a means for professional development, communities of practice arise from a common problem in the educational community, and the participating teachers interact in a continuous and committed manner (Vangrieken et al., 2017).

The three experiences, which focused on having teachers develop lesson plans that fostered students’ self-regulation, all reported positive outcomes. This appears to highlight the importance and positive impact of all of these characteristics that constitute teacher collaboration and how collaboration can promote professional development and improvement in the teaching process (Borko, 2004).

This observation was reinforced by the study from Boulahoual et al. (2024), who implemented an online course with two cohorts of student teachers. In satisfaction questionnaires, 63% of students expressed a preference for collaborative activities over individual ones, and 59% positively valued continuous assessments.

The importance of feedback and collaboration can also be seen in the study by Ambinintsoa and Castro (2024). The two participants interviewed two years after the course highlighted the value of time spent in group discussion, not only for problem-solving but also for realizing they were not the only ones encountering difficulties. They also emphasized how feedback they received from the instructors helped them learn from their own mistakes and readjust their action plans, thereby self-regulating their learning.

Secondly, some training programs appeared to face challenges in meeting all the predefined criteria. These studies by Barz et al. (2024) and Dignath-van Ewijk et al. (2015), both of which involved student teachers and pointed towards lower impact outcomes. The authors attributed this result to deficient design of the study material, characterized by excessive information (Barz et al., 2024). They also suggested that the low student motivation and interest, since it was a compulsory course, may have prevented significant gains (Barz et al., 2024; Dignath-van Ewijk et al., 2015). In this regard, Lipowsky and Rzejak (2015) concurred that motivation is an important factor in predicting the quality of learning and the effectiveness of training.

These motivational factors could be related to the low impact observed in Dignath’s (2021) program. Of the 45 teachers who participated in the 8 h course, only 16 were willing to associate their scores with those of their students, suggesting low commitment to the training. Lipowsky and Rzejak (2015) noted that it was teachers with goal-oriented teaching perspectives who most often reflected on classroom interactions, leading them to participate in professional development out of an interest in self-improvement. Therefore, internal motivational factors appear to play a significant role in the success of the interventions.

Another explanation for these low outcomes, according to Desimone (2009), may have been how long the training lasted. This author indicated that programs lasting more than 20 h or taking place throughout a semester yielded better results. However, this hypothesis appears, to some extent, to disagree with the results of the present review. While Dignath (2021) reported an 8 h program with what seems to exhibit lower results, Sins et al. (2024) and Heirweg et al. (2022), whose programs lasted 20 h or more, also suggested limited effects. Barz et al. (2024), Bruna et al. (2023), and Dignath-van Ewijk et al. (2015) were also in the group with lower impact results and measured their programs in weeks, preventing direct comparison. In contrast, Stephenson et al. (2024) implemented a course with student teachers lasting only 3 h that pointed towards very positive results, hinting that longer programs do not necessarily mean better results. As Lipowsky and Rzejak (2015) noted, what matters is what happens during instruction: the type of activities, the ways of interacting, and the learning opportunities, rather than how long the training lasts.

Heirweg et al. (2022) attributed their intervention’s smaller effects to, among other things, a failure to not specifically and explicitly address teachers’ beliefs about self-regulated learning. They suggested that perhaps changes in teachers’ self-efficacy and beliefs manifest later, once they see the effects of the intervention on their students. According to Guskey (2002), sufficient time must elapse for training participants to adapt to the newly acquired ideas and begin implementing them in their practice, making it essential to measure progress at different time intervals. Despite this recommendation, only 3 of the 30 manuscripts included a follow-up measure. These measures not only confirmed that the skills and knowledge acquired during training were maintained over time, but that they could even increase without the need for additional instruction, as shown by Michalsky (2020).

Finally, both Guskey (2002) and Lipowsky and Rzejak (2015) agreed that student outcomes are an important factor when assessing the effectiveness of a teacher training program. Lipowsky and Rzejak (2015) thought that teachers’ professional competence largely depended on their ability to evaluate their instruction and its impact on their students. If teachers see their students benefit from the professional training they themselves are getting, they will be more effective and motivated when continuing to apply this new knowledge in their teaching, leading to sustained change over time (Ryan & Deci, 2002).

Therefore, student evaluation is another pending task, since only 4 of the interventions took this effect into account. Of those, only Dignath (2021) and Heirweg et al. (2022) reported low impact results, possibly due to the aforementioned factors such as lack of teacher motivation or failure to address teacher beliefs causing lower self-confidence when teachers applied what they had learned about self-regulated learning with their own students.

## 5. Conclusions and Lines for Future Research

In this review, quantitative and qualitative studies focused on training in-service and pre-service teachers in self-regulated learning were compiled to verify its efficacy and impact, as well as the characteristics that are potentially more beneficial during the intervention. Overall, training in self-regulated learning had a positive impact on all participants’ knowledge, beliefs, and practice. Although due to the number of selected studies (n = 30) and varied characteristics, it was difficult to establish common features that could be considered key to the training’s effectiveness. Nonetheless, some factors were particularly important, such as collaboration among teachers, using videos of real classes as support, and feedback from instructors. Considering this, it could be interesting for future research to establish a standardized classification of training methods and methodologies, as well as training duration to facilitate future comparisons and identify the most effective strategies.

On the other hand, some of the questions posed at the beginning of this review, which appeared important in determining the final effectiveness of an intervention, were not fully resolved because few studies evaluated the impact of this new knowledge on students. Therefore, it is not possible to say that the training served to change teaching practice toward a more self-regulated approach. Similarly, not many studies included long-term measures to evaluate how the new knowledge and beliefs developed over time, despite how important time is for consolidating new learning.

Because of that, future lines of research should focus on ensuring knowledge of self-regulated learning in interventions by taking follow-up measures after courses finish (six months to one year) using assessment tools such as validated scales, professional evaluations or classroom observation. In the same line, interventions should start checking whether the students notice any changes in their teachers’ practice or if their learning is impacted in any way. Furthermore, the efficacy of interventions seems to be particularly founded on the participating teachers’ motivational factors. Therefore, it would be interesting to consider teachers’ motivations and interests when they take part in these training sessions, as that might be the key to an intervention producing positive results.

## 6. Limitations

Despite the study following the established methodology for systematic reviews and attempting to ensure the quality of the criteria and manuscripts included within it, there are some limitations that could have affected the results. First, no formal protocol was followed or compiled before the review process started. The inclusion criteria and the keyword search may have influenced the results and they may not have captured all of studies that could have been of interest, especially considering the potential selection bias arising from the limited set of search terms and the specific ten-year timeframe applied to the literature search. Similarly, only including articles in English, and the decision to exclude book chapters, theses, conference proceedings, and gray literature, means that the compilation presented here is not complete and may be potentially bias. The included manuscripts generally exhibit a low risk of bias, providing transparent descriptions of their methodologies and psychometric properties. However, it is recognized that potential errors or missing information in the primary studies may have remained undetected, which could affect the interpretation of the findings. Similarly, the rubric designed for the impact evaluation of the intervention, even though it is based on recognized model of professional competence, has not been validated and the results yield from it might not be reliable. In addition, the method of analysis and the portion of the work that was carried out by a single person, albeit in constant consultation with experts in the field, mean that the risk of misinterpretation or inadvertent oversight of relevant details or data cannot be entirely dismissed. These factors could affect the generalization of the results found, and in future studies, a multi-researcher extraction should be implemented to enhance reliability. These limitations are inherent to the nature of systematic qualitative synthesis

Finally, as stated on the conclusions and lines for future research, the findings regarding the long-term impact of the training on teachers or on their students cannot be generalized, given the limited number of professional development programs that accounted for these variables.

## Figures and Tables

**Table 1:** Inclusion and exclusion criteria.

Criteria	Inclusion	Exclusion
Sample	Active teachers working at any educational level and pre-service teachers (Education degree students or Teacher Training Master’s students).	Students in Primary Education, Secondary Education or any degree or master’s degree not related to the future teaching profession.
Phenomenon of interest	Self-Regulated training (MOOC courses, webinars, face-to-face courses, etc.).	Studies that analyze the development of technical skills without explicitly addressing self-regulation.
Design	Experimental, quasi-experimental or qualitative studies.	Observational or descriptive studies that do not include any training.
Evaluation	Studies that analyze the development of SRL in teachers and/or studies that assess the impact of the SRL training on teachers performance.	Studies that do not measure SRL related outcomes in in-service or pre-service teachers.
Research type	Intervention studies (research that observes the effect of training programs on teacher SRL) and/or program evaluation studies (research that evaluates the effectiveness and impact of SRL training programs).	Theoretical studies, book chapters or doctoral theses.

**Table 2:** Methodological quality indicators (MQIs).

Standard	Quality Criteria
1. Provides clear argument that links theory and research in a way that builds the formulation of the question (s)	1.1 The study provides clear rationale and links previous research to the formulation of research questions or objectives. 1.2 Findings are explicitly linked to existing theory, prior research or scholarly arguments
2. Applies rigorous, systematic, and objective methodology to obtain reliable and valid knowledge relevant to educational activities and programs	2.1 Methods are described with sufficient clarity to allow for study replication, including detailed data collection and analysis procedures. 2.2 Evidence of reliability is provided (e.g., Cronbach’s alpha for quantitative data or credibility/trustworthiness for qualitative designs). 2.3 Validity: The study demonstrates that instruments or data collection tools accurately measure the intended constructs. 2.4 Participants and research contexts are well-defined (e.g., age, educational level, and subject area).
3. Presents findings and makes claims that are supported by the methods employed	3.1 Conclusions and claims are legitimate, directly supported by the collected data, and consistent with the results.

Source: Miller et al. (2016).

**Table 3:** Data extraction coding tool.

Category	Elements
Paper general characteristics	- Authors. - Publication Year. - Methodological Design. - Sample (in-service or pre-service). - Number of participants. - Country.
General characteristics of the training program	- Type of training/program. - Training modality (online or face-to-face). - Pedagogical approach. - SRL components included. - Duration of the training.
Evaluation of the training program	- Training outcomes. - Measurement times. - Evaluation of teaching practice (yes/no) and indicators used. - Evaluation of students (yes/no), instruments used and results obtained. - Longitudinal follow-up (yes/no), duration of the follow-up and effects found.

## Data Availability

Not applicable.

## Supplementary Materials

The following supporting information can be downloaded at: https://www.mdpi.com/article/10.3390/ejihpe16040055/s1, PRISMA checklist.

## Appendix A

**Table A1:** Quality assessment of the studies included in the review.

Reference	1.1. Explain Theory	1.2. Links Results-Theory	2.1. Methods	2.2. Reliability	2.3. Validity	2.4. Participants	3.1. Results	Total
Adigüzel et al. (2023)	1	1	1	1	1	1	1	7
Ambinintsoa and Castro (2024)	1	1	1	1	1	1	1	7
Barr and Askell-Williams (2020)	1	1	1	1	1	1	1	7
Bruna et al. (2023)	1	1	1	1	1	1	1	7
Cleary et al. (2022)	1	1	1	1	1	1	1	7
Dignath (2021)	1	1	1	1	1	1	1	7
Heaysman and Kramarski (2022)	1	1	1	1	1	1	1	7
Heirweg et al. (2022)	1	1	1	1	1	1	1	7
Linde et al. (2023)	1	1	1	0	1	1	1	6
Persico et al. (2023)	1	1	1	0	1	1	1	6
Peters-Burton et al. (2024)	1	1	1	1	1	1	1	7
Porter and Peters-Burton (2021)	1	1	1	1	1	1	1	7
Sins et al. (2024)	1	1	1	1	1	1	1	7
Xu and Ko (2019)	1	1	1	1	1	1	1	7
Arcoverde et al. (2020)	1	1	1	1	1	1	1	7
Barz et al. (2024)	1	1	1	1	1	1	1	7
Dignath-van Ewijk et al. (2015)	1	1	1	1	1	1	1	7
Ganda and Boruchovitch (2018)	1	1	1	1	1	1	1	7
Hursen and Fasli (2017)	1	1	1	1	1	1	1	7
Inan-Karagul and Seker (2021)	1	1	1	1	1	1	1	7
Kaemper et al. (2024)	1	1	1	1	1	1	1	7
Kramarski and Kohen (2017)	1	1	1	1	1	1	1	7
Michalsky (2014)	1	1	1	1	1	1	1	7
Michalsky (2020)	1	1	1	1	1	1	1	7
Michalsky (2024)	1	1	1	1	1	1	1	7
Ortega-Ruiperez et al. (2024)	1	1	1	1	1	0	1	6
Romig et al. (2018)	1	1	1	1	1	1	1	7
Stephenson et al. (2024)	1	1	1	1	1	1	1	7
Tran et al. (2022)	1	1	1	1	1	1	1	7
Zeeb et al. (2024)	1	1	1	1	1	1	1	7

## Appendix B

**Table A2:** Characteristics of the review studies.

Author/Year	Design	Sample	Results	Ev. Practice	Ev. Students	Follow-Up	Intervention	Method	Theory	SRL Component	Duration
In-service											
Adigüzel et al. (2023)	Mix, case study	80, Turkey	High	Lesson plan/records	Increase		Learning community	Online	Zimmerman	Meta/cognition/emotional	16 weeks
Ambinintsoa and Castro (2024)	Case study	2 cases, Madagascar	High	Interview	No	2 years	Diaries and discussion groups	Face2face	Zimmerman	Reflective/self assessment	9 weeks
Barr and Askell-Williams (2020)	Microgenetics	4, Australia	High	Lesson plan	No		Learning community	Face2face	Zimmerman/3R-EC	Knowledge/beliefs	12 weeks
Bruna et al. (2023)	Quasi-experimental	60, Chile	Low	No	No		Course	Face2face	Zimmerman	Reflection, design, assessment, efficacy	8 weeks
Cleary et al. (2022)	Mix sequential	teachers	Moderate	Lesson Plan	No		Course	Face2face	Zimmerman	SRL, self-efficacy, application	1 week
Dignath (2021)	Quasi-experimental	45, Germany	Low	Yes	No changes		Workshop	Face2face	Zimmerman y Boeakert	Beliefs, self-efficacy, cognitive approach	8 h
Heaysman and Kramarski (2022)	Quasi-experimental	76, Israel	Moderate	Lesson Plan	No		AIDE:video and simulations	Face2face	Zimmerman	Identification and application strategies	30 h
Heirweg et al. (2022)	Quasi-experimental	40, Belgium	Low	Questionnaire	Slightly		Course	Face2face	Zimmerman	Direct and indirect instruction	27.5 h
Linde et al. (2023)	Mix	103, Latvia	Moderate	Yes	No		Course	online	Zimmerman	3 phases SRL	1 year
Persico et al. (2023)	Exploratory study	8, Italy, 4, Belgium y 2, Spain	Moderate	No	No		Board game	Face2face	Zimmerman	Self-competency SRL and beliefs	3 days
Peters-Burton et al. (2024)	Mix	19, USA	Moderate	No	No		Digital tool	Hybrid (pandemic)	Zimmerman	SRL	3 years
Porter and Peters-Burton (2021)	Multiple cases	9, USA	Moderate	Lesson plan/worksheet	No		Course	Face2face	Zimmerman	Activity design	32 weeks
Sins et al. (2024)	Quasi-experimental	32, Netherlands	Low	lesson Plan/observation	No		Digital tool	online	Zimmerman y Pintrich	Explicit teaching	20 h
Xu and Ko (2019)	Case study	11, China	High	records and materials	Increase		Community of practice	Face2face		SRL theory and lesson plan design	6–11 h
Pre-service											
Arcoverde et al. (2020)	Quasi-experimental	220, Brazil	Moderate	No	No		Focus group and lesson plan	Face2face	Zimmerman	Strategies	5 weeks
Barz et al. (2024)	Profile analysis	145, Germany	Low	No	No		synchronous and asynchronous	Online	Zimmerman	Declarative knowledge	6 weeks
Dignath-van Ewijk et al. (2015)	Quasi-experimental	65, Germany	Low	No	No		Learning diaries	Online	Zimmerman	Strategies and skills	13 weeks
Ganda and Boruchovitch (2018)	Experimental	109, Brazil	Moderate	No	No		Intervention program vs course	Face2face	Zimmerman	Strategies, self-efficacy and beliefs	12 h
Hursen and Fasli (2017)	Case study	36, Cyprus	High	No	No		Edmodo	Online		Reflection and self directed learning	12 weeks
Inan-Karagul and Seker (2021)	Qualitative	135, Turkey	High	No	No		Screencast feedback	online	Zimmerman	Strategies and writing	6 weeks
Kaemper et al. (2024)	Quasi-experimental	95, Germany	Moderate	No	No		Digital tool	online	Zurich resources model	Self-reflection	2 weeks
Kramarski and Kohen (2017)	Case study	90, Israel	High	No	No		Seminar, video and prompts	Face2face	Zimmerman y Pintrich	Metacognition and motivation	14 seminars
Michalsky (2014)	Case study, quantitative	26, Israel	Moderate	No	No		Course and videos	Face2face	Boekaerts	Direct and indirect teaching	12 weeks
Michalsky (2020)	Quasi-experimental	102, Israel	High	No	No	1 semester	Videos	online	Boekaerts	Direct and indirect teaching and strategies	12 weeks
Michalsky (2024)	Mix exploratory	67, Israel	Moderate	No	No		Digital tool/scaffolding	online	Zimmerman	Metacognition	14 weeks
Ortega-Ruiperez et al. (2024)	Quasi-experimental	242, Spain	Moderate	No	No		Digital tool	hybrid	Zimmerman	Cognition, motivation, compression, planification, environment	
Romig et al. (2018)	Experimental	166, USA	Moderate	Videos	No		Podcast, article and lecture	Face2face		knowledge	One day
Stephenson et al. (2024)	Qualitative	91, Australia	High	No	No	6 weeks	Course			SRT	3 h
Tran et al. (2022)	Qualitative	12, USA	High	Practicum	No		Course	Face2face	Zimmerman	SRL	2 weeks
Zeeb et al. (2024)	Quasi-experimental	119, Germany	Moderate	No	No		Course	Face2face	Zimmerman	Declarative knowledge, self-reported and feedback	12 seminars

## Appendix C

**Table A3:** Scores of the studies based on the Baumert and Kunter (2013) professional competence definition.

	Knowledge and Skills		Beliefs		Motivation		SRL
Authors	Did Knowledge About SRL Improve?	Did Knowledge About How to Intervene Improve?	Did Perceptions and Conceptions About SRL Change?	Is There More Reflection on One’s Own Teaching Practice?	Did Interest in SRL Increase?	Did the Perception of the Benefits of SRL Increase?	Did Teacher Self-Efficacy Increase?
In-service							
Adigüzel et al. (2023)	3	3	Not applicable	3	Not applicable	3	3
Ambinintsoa and Castro (2024)	3	3	3	3	3	3	3
Barr and Askell-Williams (2020)	2	3	3	3	Not applicable	Not applicable	3
Bruna et al. (2023)	0	3	1	1	Not applicable	1	2
Cleary et al. (2022)	2	3	2	Not applicable	Not applicable	3	3
Dignath (2021)	1	2	1	Not applicable	Not applicable		2
Heaysman and Kramarski (2022)	2	3	3	2	Not applicable	2	3
Heirweg et al. (2022)	1	2	0	Not applicable	1	Not applicable	0
Linde et al. (2023)	3	2	2	Not applicable	Not applicable	3	2
Persico et al. (2023)	2	2	0	Not applicable	3	3	2
Peters-Burton et al. (2024)	2	2	3	3	3	3	2
Porter and Peters-Burton (2021)	2	3	1	1	Not applicable	2	3
Sins et al. (2024)	2	1	Not applicable	2	Not applicable	3	1
Xu and Ko (2019)	3	3	2	3	2	3	2
Pre-service							
Arcoverde et al. (2020)	2	Not applicable	2	2	2	3	2
Barz et al. (2024)	2	3	3	3	Not applicable	Not applicable	3
Dignath-van Ewijk et al. (2015)	1	2	Not applicable	2	0	2	1
Ganda and Boruchovitch (2018)	2	Not applicable	Not applicable	2	2	2	2
Hursen and Fasli (2017)	2	3	Not applicable	3	3	3	3
Inan-Karagul and Seker (2021)	2	2	3	3	3	3	3
Kaemper et al. (2024)	2	Not applicable	Not applicable	3	2	2	Not applicable
Kramarski and Kohen (2017)	2	3	Not applicable	Not applicable	3	3	3
Michalsky (2014)	2	3	Not applicable	2	Not applicable	2	Not applicable
Michalsky (2020)	3	3	Not applicable	2	2	3	3
Michalsky (2024)	3	2	Not applicable	3	Not applicable	Not applicable	2
Ortega-Ruiperez et al. (2024)	2	2	Not applicable	2	Not applicable	2	2
Romig et al. (2018)	2	2	Not applicable	Not applicable	Not applicable	3	2
Stephenson et al. (2024)	3	2	2	Not applicable	Not applicable	3	3
Tran et al. (2022)	3	2	Not applicable	3	3	3	2
Zeeb et al. (2024)	3	3	Not applicable	2	Not applicable	Not applicable	1

## References

[B1-ejihpe-16-00055] Adigüzel T., Asik G., Bulut M. A., Kaya M. H., Özel S. (2023). Teaching self-regulation through role modeling in K-12. Frontiers in Education.

[B2-ejihpe-16-00055] Aguilera R. (2014). ¿Revisión sistemática, revisión narrativa o metaanálisis?. Revista de la Sociedad Española del Dolor.

[B3-ejihpe-16-00055] Ambinintsoa D. V., Castro E. (2024). Self-regulation to develop autonomy in language teacher education: Two case studies in an EFL Malagasy context. AILA Review.

[B4-ejihpe-16-00055] Amemasor S. K., Oppong S. O., Ghansah B., Benuwa B.-B., Essel D. D. (2025). A systematic review on the impact of teacher professional development on digital instructional integration and teaching practices. Frontiers in Education.

[B5-ejihpe-16-00055] Arcoverde A. R. dos R., Boruchovitch E., Acee T. W., Goes N. M. (2020). Self-regulated learning of Brazilian students in a teacher education program in Piaui: The impact of a self-regulation intervention. Frontiers in Education.

[B6-ejihpe-16-00055] Barr S., Askell-Williams H. (2020). Changes in teachers’ epistemic cognition about self-regulated learning as they engaged in a researcher-facilitated professional learning community. Asia-Pacific Journal of Teacher Education.

[B7-ejihpe-16-00055] Barz N., Benick M., Dörrenbächer-Ulrich L., Perels F. (2024). Fostering self-regulated learning using synchronous or asynchronous digital learning environments: A latent profile analysis of pre-service teachers’ individual differences. Frontiers in Education.

[B8-ejihpe-16-00055] Baumert J., Kunter M., Kunter M., Baumert J., Blum W., Klusmann U., Krauss S., Neubrand M. (2013). The COACTIV model of teachers’ professional competence. Cognitive activation in the mathematics classroom and professional competence of teachers results from the COACTIV project.

[B9-ejihpe-16-00055] Bautista A., Ortega-Ruíz R. (2015). Teacher professional development: International perspectives and approaches. Psychology, Society & Education.

[B10-ejihpe-16-00055] Boekaerts M. (1997). Self-regulated learning: A new concept embraced by researchers, policy makers, educators, teachers, and students. Learning and Instruction.

[B11-ejihpe-16-00055] Borko H. (2004). Professional development and teacher learning: Mapping the terrain. Educational Researcher.

[B12-ejihpe-16-00055] Boulahoual A., Ouasri A., Abid M., el Mahjoub C. (2024). New digital learning model for srl-oriented instructional design and flexibility. International Journal on Technical and Physical Problems of Engineering.

[B13-ejihpe-16-00055] Brock M. E., Carter E. W. (2017). A meta-analysis of educator training to improve implementation of interventions for students with disabilities. Remedial and Special Education: RASE.

[B14-ejihpe-16-00055] Bruna D., Pérez M. V., Bustos C., Villarroel V. (2023). The impact of a university teacher training program promoting self-regulated learning on teacher knowledge, self-efficacy, and practices. Frontiers in Education.

[B15-ejihpe-16-00055] Callan G. L., Shim S. S. (2019). How teachers define and identify self-regulated learning. The Teacher Educator.

[B16-ejihpe-16-00055] Camacho A., Alves R. A., Boscolo P. (2021). Writing motivation in school: A systematic review of empirical research in the early twenty-first century. Educational Psychology Review.

[B17-ejihpe-16-00055] Chu L., Li P.-H., Yu M.-N. (2020). The longitudinal effect of children’s self-regulated learning on reading habits and well-being. International Journal of Educational Research.

[B18-ejihpe-16-00055] Cleary T. J., Kitsantas A., Peters-Burton E., Lui A., McLeod K., Slemp J., Zhang X. (2022). Professional development in self-regulated learning: Shifts and variations in teacher outcomes and approaches to implementation. Teaching and Teacher Education.

[B19-ejihpe-16-00055] Darling-Hammond L., Hyler M. E., Gardner M. (2017). Effective teacher professional development.

[B20-ejihpe-16-00055] de Boer H., Donker A. S., Kostons D. D. N. M., van der Werf G. P. C. (2018). Long-term effects of metacognitive strategy instruction on student academic performance: A meta-analysis. Educational Research Review.

[B21-ejihpe-16-00055] Desimone L. M. (2009). Improving impact studies of teachers’ professional development: Toward better conceptualizations and measures. Educational Researcher.

[B22-ejihpe-16-00055] Desimone L. M., Garet M. S. (2015). Best practices in teachers’ professional development in the United States. Psychology, Society and Education.

[B23-ejihpe-16-00055] Dignath C. (2021). For unto everyone that hath shall be given: Teachers’ competence profiles regarding the promotion of self-regulated learning moderate the effectiveness of short-term teacher training. Metacognition and Learning.

[B24-ejihpe-16-00055] Dignath-van Ewijk C., Büttner G. (2018). Teachers’ direct and indirect promotion of self-regulated learning in primary and secondary school mathematics classes. Metacognition and Learning.

[B25-ejihpe-16-00055] Dignath-van Ewijk C., Fabriz S., Buttner G. (2015). Fostering self-regulated learning among students by means of an electronic learning diary: A training experiment. Journal of Cognitive Education and Psychology.

[B26-ejihpe-16-00055] Dignath-van Ewijk C., van der Werf G. (2012). What teachers think about self regulated learning: Investigating teacher beliefs and teacher behavior of enhancing students’ self-regulation. Educational Research International.

[B27-ejihpe-16-00055] Donath J. L., Lüke T., Graf E., Tran U. S., Götz T. (2023). Does professional development effectively support the implementation of inclusive education? A meta-analysis. Educational Psychology Review.

[B28-ejihpe-16-00055] Ganda D. R., Boruchovitch E. (2018). Promoting self-regulated learning of Brazilian preservice student teachers: Results of an intervention program. Frontiers in Education.

[B29-ejihpe-16-00055] Guskey T. R. (2002). Does it make a difference? Evaluating professional development. Educational Leadership.

[B30-ejihpe-16-00055] Heaysman O., Kramarski B. (2022). Promoting teachers’ in-class SRL practices: Effects of Authentic Interactive Dynamic Experiences (AIDE) based on simulations and video. Instructional Science.

[B31-ejihpe-16-00055] Heirweg S., de Smul M., Merchie E., Devos G., van Keer H. (2022). The long road from teacher professional development to student improvement: A school-wide professionalization on self-regulated learning in primary education. Research Papers in Education.

[B32-ejihpe-16-00055] Hursen C., Fasli F. G. (2017). The impact of reflective teaching applications supported by Edmodo on prospective teachers’ self-directed learning skills. International Journal of Emerging Technologies in Learning.

[B33-ejihpe-16-00055] Inan-Karagul B., Seker M. (2021). Improving language learners’ use of self-regulated writing strategies through screencast feedback. SAGE Open.

[B34-ejihpe-16-00055] Kaemper M., Buhl H. M., Klingsieck K. B. (2024). How to improve student teachers’ self-regulated competency development: Effects of a resource-oriented training program. Zeitschrift Für Entwicklungspsychologie Und Pädagogische Psychologie.

[B35-ejihpe-16-00055] Kalinowski E., Gronostaj A., Vock M. (2019). Effective professional development for teachers to foster students’ academic language proficiency across the curriculum: A systematic review. AERA Open.

[B36-ejihpe-16-00055] Kramarski B., Kohen Z. (2017). Promoting preservice teachers’ dual self-regulation roles as learners and as teachers: Effects of generic vs. specific prompts. Metacognition and Learning.

[B37-ejihpe-16-00055] Kyndt E., Gijbels D., Grosemans I., Donche V. (2016). Teachers’ everyday professional development: Mapping informal learning activities. Teaching and Teacher Education.

[B38-ejihpe-16-00055] Landis J. R., Koch G. G. (1977). The measurement of observer agreement for categorical data. Biometrics.

[B39-ejihpe-16-00055] Latva-aho J., Näykki P., Pyykkönen S., Laitinen-Väänänen S., Hirsto L., Veermans M. (2024). Pre-service teachers’ ways of understanding, observing, and supporting self-regulated learning. Teaching and Teacher Education.

[B40-ejihpe-16-00055] Lay C. D., Allman B., Cutri R. M., Kimmons R. (2020). Examining a decade of research in online teacher professional development. Frontiers in Education.

[B41-ejihpe-16-00055] Linde I., Sarva E., Daniela L. (2023). The impact of an online professional development course on teachers’ comprehension and self-efficacy in developing students’ self-regulated learning skills. Sustainability.

[B42-ejihpe-16-00055] Lipowsky F., Rzejak D. (2015). Key features of effective professional development programmes for teachers. Ricercazione.

[B43-ejihpe-16-00055] Michalsky T. (2014). Developing the SRL-PV assessment scheme: Preservice teachers’ professional vision for teaching self-regulated learning. Studies in Educational Evaluation.

[B44-ejihpe-16-00055] Michalsky T. (2020). Preservice teachers’ professional vision for and capacity to teach self-regulated learning: Effects of scaffolding level. Teachers College Record.

[B45-ejihpe-16-00055] Michalsky T. (2024). Metacognitive scaffolding for preservice teachers’ self-regulated design of higher order thinking tasks. Heliyon.

[B46-ejihpe-16-00055] Miller D. M., Scott C. E., McTigue E. M. (2016). Writing in the secondary-level disciplines: A systematic review of context, cognition, and content. Educational Psychology Review.

[B47-ejihpe-16-00055] Ortega-Ruiperez B., Pereles A., Lazaro M. (2024). Impact of a digital tool to improve metacognitive strategies for self-regulation during text reading in online teacher education. Journal of Information Technology Education-Innovations in Practice.

[B48-ejihpe-16-00055] Page M. J., McKenzie J. E., Bossuyt P. M., Boutron I., Hoffmann T. C., Mulrow C. D., Shamseer L., Tetzlaff J. M., Akl E. A., Brennan S. E., Chou R. (2021). The PRISMA 2020 statement: An updated guideline for reporting systematic reviews. BMJ.

[B49-ejihpe-16-00055] Panadero E. (2017). A review of self-regulated learning: Six models and four directions for research. Frontiers in Psychology.

[B50-ejihpe-16-00055] Panadero E., Alonso-Tapia J. (2014). ¿Cómo autorregulan nuestros alumnos? Modelo de Zimmerman sobre estrategias de aprendizaje. Anales de Psicología.

[B51-ejihpe-16-00055] Perry N. E., Hutchinson L., Thauberger C. (2008). Talking about teaching self-regulated learning: Scaffolding student teachers’ development and use of practices that promote self-regulated learning. International Journal of Educational Research.

[B52-ejihpe-16-00055] Perry N. E., VandeKamp K. J. O. (2000). Creating classroom contexts that support young children’s development of self-regulated learning. International Journal of Educational Research.

[B53-ejihpe-16-00055] Persico D., Manganello F., Passarelli M., Pozzi F. (2023). Is GBL good for teachers? A game for teachers on how to foster students’ self-regulated learning. Education Sciences.

[B54-ejihpe-16-00055] Peters-Burton E. E., Tran H. H., Miller B. (2024). Design-based research as professional development: Outcomes of teacher participation in the development of the science practices innovation notebook (SPIN). Journal of Science Teacher Education.

[B55-ejihpe-16-00055] Pintrich P. R., Wolters C. A., Baxter G. P., Schraw G., Impara J. (2000). Assessing metacognition and self-regulated learning. Issues in the measurement of metacognition.

[B56-ejihpe-16-00055] Porter A. N., Peters-Burton E. E. (2021). Investigating teacher development of self-regulated learning skills in secondary science students. Teaching and Teacher Education.

[B57-ejihpe-16-00055] Romig J. E., Sundeen T., Thomas C. N., Kennedy M. J., Philips J., Peeples K. N., Rodgers W. J., Mathews H. M. (2018). Using multimedia to teach self-regulated strategy development to preservice teachers. Journal of Special Education Technology.

[B58-ejihpe-16-00055] Ryan R. M., Deci E. L., Ryan R. M., Deci E. L. (2002). Overview of self-determination theory: An organismic dialectical perspective. Handbook of self-determination research.

[B59-ejihpe-16-00055] Sankar L., Sankar C. S. (2010). Comparing the effectiveness of face-to-face and online training on teacher knowledge and confidence. Proceedings of informing science & IT education conference (InSITE).

[B60-ejihpe-16-00055] Sánchez-Martín M., Pedreño M., Ponce A. I., Navarro-Mateu F. (2023). Y, al principio, fue la pregunta de investigación … Los formatos PICO, PECO, SPIDER y FINER [And, at first, it was the research question… The PICO, PECO, SPIDER and FINER formats]. Espiral. *Cuadernos del Profesorado*.

[B61-ejihpe-16-00055] Siegle D., McCoach D. B. (2007). Increasing student mathematics self-efficacy through teacher training. Journal of Advanced Academics.

[B62-ejihpe-16-00055] Sins P., de Leeuw R., de Brouwer J., Vrieling-Teunter E. (2024). Promoting explicit instruction of strategies for self-regulated learning: Evaluating a teacher professional development program in primary education. Metacognition and Learning.

[B63-ejihpe-16-00055] Stavermann K. (2024). Online teacher professional development: A research synthesis on effectiveness and evaluation. Technology Knowledge and Learning.

[B64-ejihpe-16-00055] Stephenson H., Lawson M. J., Nguyen-Khoa L.-A., Kang S. H. K., Vosniadou S., Murdoch C., Graham L., White E. (2024). Helping teacher education students’ understanding of self-regulated learning and how to promote self-regulated learning in the classroom. Frontiers in Education.

[B65-ejihpe-16-00055] Theobald M. (2021). Self-regulated learning training programs enhance university students’ academic performance, self-regulated learning strategies, and motivation: A meta-analysis. Contemporary Educational Psychology.

[B66-ejihpe-16-00055] Tran H. H., Capps D. K., Hodges G. W. (2022). Preservice science teachers’ perspectives on and practices related to self-regulated learning after a brief learning opportunity. Sustainability.

[B67-ejihpe-16-00055] Valiente-Barroso C., Suárez-Riveiro J. M., Martínez-Vicente M. (2020). Autorregulación del aprendizaje, estrés escolar y rendimiento académico. European Journal of Education and Psychology.

[B68-ejihpe-16-00055] Vangrieken K., Meredith C., Packer T., Kyndt E. (2017). Teacher communities as a context for professional development: A systematic review. Teaching and Teacher Education.

[B69-ejihpe-16-00055] Xu H., Ko P. Y. (2019). Enhancing teachers’ knowledge of how to promote self-regulated learning in primary school students: A case study in Hong Kong. Teaching and Teacher Education.

[B70-ejihpe-16-00055] Zeeb H., Bürgermeister A., Saalbach H., Renkl A., Glogger-Frey I. (2024). Effects of a digital support tool on student teachers’ knowledge about, assessment of, and feedback on self-regulated learning. Unterrichtswissenschaft.

[B71-ejihpe-16-00055] Zimmerman B. J., Boekaerts M., Pintrich P. R., Zeidner M. (2000). Attaining self-regulation: A social cognitive perspective. Handbook of self-regulation.

